# Type 2 diabetes mellitus – conventional therapies and future perspectives in innovative treatment

**DOI:** 10.1016/j.bbrep.2025.102037

**Published:** 2025-05-02

**Authors:** Barbara Gieroba, Adrianna Kryska, Anna Sroka-Bartnicka

**Affiliations:** Medical University of Lublin, Independent Unit of Spectroscopy and Chemical Imaging, Chodzki 4a, 20-093, Lublin, Poland

**Keywords:** Glucose metabolism regulation, Diabetes management strategies, Insulin resistance, Novel drug targets, Emerging drugs in T2DM

## Abstract

Type 2 Diabetes Mellitus (T2DM) is a chronic metabolic disorder characterized by insulin resistance and dysfunction of the pancreatic beta cells, which leads to elevated blood glucose levels. Conventional therapies, including metformin, sulfonylureas, and insulin, have long served as the cornerstone of treatment. However, they often face limitations, such as adverse effects, reduced efficacy over time, and difficulties in achieving optimal glycemic control. This has sparked considerable interest in developing novel and experimental therapeutic strategies to enhance treatment outcomes. Recent advances in diabetes management feature dual incretin receptor agonists, like tirzepatide, which combine GLP-1 and GIP receptor agonism, resulting in increased insulin secretion, decreased glucagon release, and significant weight loss. Additionally, dual SGLT1/2 inhibitors, such as sotagliflozin, show promise for more significant blood glucose reduction and improved weight loss by targeting glucose regulation in both the gut and kidneys. Other promising methods include glucagon receptor antagonists, GPR119 agonists, and FGF21 analogs, which strive to enhance insulin sensitivity and improve glucose metabolism through innovative pathways. Gene editing technologies, including CRISPR-Cas9 and AMPK activators, are also being investigated to tackle the underlying pathophysiology of T2DM more effectively. While these experimental therapies show promise, their long-term safety and efficacy remain under research. This article reviews the conventional therapies currently in use. It investigates future perspectives on innovative treatments for T2DM, emphasizing the potential of these new therapies to transform diabetes care and enhance patient outcomes.

## Highlights

• Metformin, sulfonylureas, and insulin are standard therapies but can cause side effects and lose efficacy over time.
• Dual incretin agonists, SGLT1/2 inhibitors, and gene-editing offer improved glucose control and stability.
• Glucagon receptor blockers, GPR119 agonists, and FGF21 analogs improve glucose use and insulin sensitivity.
• Combining new therapies with personalized care may transform diabetes management and outcomes.

## Graphical abstract

**Figure:**
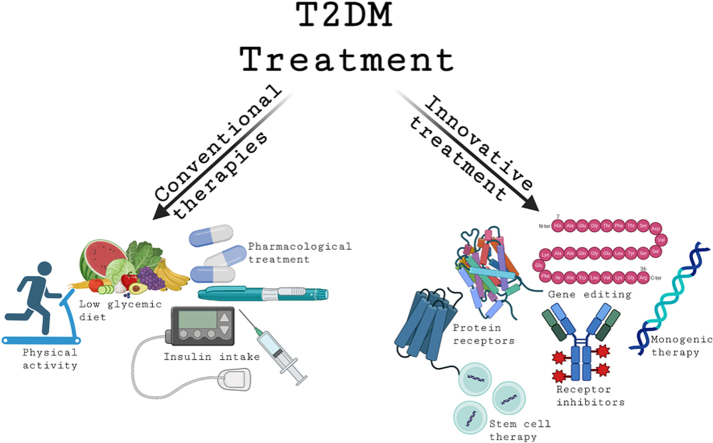

## 1 Introduction

### 1.1 Epidemiology - global prevalence and Incidence

Type 2 Diabetes Mellitus (T2DM) has become a significant public health concern, affecting millions globally, and has been rising dramatically worldwide. Importantly, T2DM accounts for over 96 % of all diabetes cases [1]. In 2021, the International Diabetes Federation (IDF) reported that global healthcare expenditures for diabetes management among individuals aged 20 to 79 reached approximately $966 billion, with projections suggesting they could exceed $1054 billion by 2045 [2]. Notably, 537 million people around the world were living with diabetes in 2021 (1 in 10 adults), representing a global age-standardized prevalence of 6.1 %. This reflects a substantial increase of 90.5 % from 3.2 % in 1990, which could rise to 783 million by 2045. The prevalence is projected to escalate to 9.8 %, potentially affecting an estimated 13.1 billion individuals. Type 2 diabetes mellitus (T2DM) is by far the most common form of diabetes, accounting for approximately 90 % of cases globally [2].

### 1.2 Pathophysiology of diabetes

T2DM is a complex metabolic disorder primarily characterized by insulin resistance (IR) and beta-cell dysfunction, which result in chronic hyperglycemia and disrupt normal glucose homeostasis. The pathophysiology of T2DM involves a combination of genetic, environmental, and lifestyle factors that collectively contribute to the disease's development and progression. Insulin resistance, which mainly affects the skeletal muscle, liver, intestines, and adipose tissues, is one of the core features of T2DM [3]. Metabolic disturbances involve elevated saturated fatty acids (SFAs) that induce adipocyte hypoxia and inflammation due to macrophage infiltration, resulting in insulin resistance (IR) and increased lipolysis. Lipolysis products, such as nonesterified fatty acids (NEFAs) and glycerol, contribute to liver fat accumulation, gluconeogenesis, and glucose release while impairing insulin signaling. Ectopic fat deposition in muscle raises circulating very low-density lipoprotein (VLDL) levels, exacerbating lipid metabolism and IR. Chronic hyperglycemia and excess fatty acids further impair β-cell function through inflammation, endoplasmic reticulum stress, and oxidative stress [4,5].

In many individuals with T2DM, there is also a buildup of islet amyloid polypeptide (IAPP) in the pancreas, contributing to beta-cell toxicity and further reducing insulin secretion [6]. Alongside these mechanisms, chronic low-grade inflammation significantly contributes to the pathophysiology of T2DM. Adipose tissue, especially visceral fat, secretes pro-inflammatory cytokines like TNF-alpha and IL-6, which promote systemic insulin resistance by disrupting insulin signaling pathways in muscle, liver, and fat tissue. These inflammatory mediators further worsen the underlying metabolic dysfunction in T2DM [7,8].

Additionally, T2DM is associated with dysregulated glucagon secretion. Usually, glucagon helps raise blood glucose by stimulating the liver to release glucose. In individuals with T2DM, alpha cells of the pancreas secrete excess glucagon, even when blood glucose levels are already elevated, which further promotes hyperglycemia by increasing hepatic glucose production [9]. Dyslipidemia is another key factor contributing to T2DM, a condition marked by elevated free fatty acids, high triglycerides, and low HDL cholesterol levels. Dyslipidemia contributes to insulin resistance and causes lipotoxicity, which damages tissues such as muscle, liver, and pancreatic beta-cells, further impairing their function [10]. Emerging research also suggests that the gut microbiota may play a role in the pathogenesis of T2DM. Alterations in the composition of gut bacteria, known as dysbiosis, can lead to increased intestinal permeability, allowing endotoxins like lipopolysaccharides to permeate the bloodstream. These endotoxins contribute to systemic inflammation and further insulin resistance [11,12].

Inflammation and oxidative stress are central to the development and progression of T2DM and its complications. Chronic hyperglycemia activates the immune system, leading to the production of pro-inflammatory cytokines and reactive oxygen species (ROS). These ROS not only cause cellular damage but also activate transcription factors like NF-κB and AP-1, which further amplify inflammation. Excess adipose tissue in diabetes adds to this process by secreting additional inflammatory mediators such as TNF-α, IL-1, and IL-6. Hyperglycemia also activates the hexosamine pathway, increasing OGT activity and altering gene expression via transcription factors like Sp1. This contributes to complications such as neuropathy (via PAI-1) and nephropathy (via TGF-β1), both of which are associated with increased ROS production [13]. In pancreatic beta cells, oxidative stress impairs insulin secretion by disrupting mitochondrial function and inducing apoptosis, thus worsening hyperglycemia and perpetuating oxidative damage. Three key biochemical pathways link ROS to diabetic complications: the aldose reductase pathway, which depletes antioxidant reserves and contributes to retinopathy; the formation of AGEs, which alter proteins and promote inflammation; and PKC activation, which drives ROS generation and vascular dysfunction. These mechanisms contribute to microvascular complications like retinopathy, nephropathy, and neuropathy by damaging blood vessels, neurons, and kidney tissue [14,15].

Ultimately, the persistent interplay between inflammation and oxidative stress creates a vicious cycle, damaging vital organs and exacerbating diabetes. Targeting these pathways may help prevent or slow the progression of diabetic complications, such as cardiovascular disease (CVD) [16].

Genetic factors are also integral to the development of T2DM. Several genes involved in insulin action, glucose metabolism, and insulin secretion have been identified as risk factors for the disease. These genetic predispositions interact with environmental factors such as diet, physical activity, and obesity to increase the likelihood of developing T2DM [17,18]. Obesity, incredibly visceral fat accumulation, is a significant risk factor for T2DM [19]. Adipose tissue is not just a storage site for fat; it is an active endocrine organ that secretes hormones and cytokines that influence insulin sensitivity. Excess visceral fat causes an imbalance in the secretion of adipokines such as leptin, adiponectin, and resistin, further impairing insulin sensitivity [20,21]. The stages of the T2DM development process are summarized in Table 1.

**Table 1:** Pathogenesis of Type 2 Diabetes (T2DM). Prepared based on [3,5,22,23].

Stage/Process	Key Mechanism/Events	Implications/Effects
1. Excess SFAs (Saturated Fatty Acids)	❖ Enhanced metabolism of SFAs in adipocytes ❖Adipocyte stress and hypoxia due to increased SFA metabolism	❖Activation of inflammatory pathways in adipose tissue ❖Adipocyte insulin resistance (IR)
2. Inflammation in Adipose Tissue	❖Hypoxia triggers macrophage infiltration and inflammatory cytokine release (e.g., TNF-α, IL-1β)	❖Chronic low-grade inflammation in adipose tissue ❖Insulin resistance in adipocytes
3. Lipolysis and Release of NEFAs (Non-Esterified Fatty Acids)	❖Insulin resistance leads to increased lipolysis ❖NEFAs and glycerol released into the bloodstream	❖Elevated lipid levels in circulation ❖Increased hepatic lipid synthesis and gluconeogenesis
4. Ectopic Fat Deposition in the Liver	❖NEFAs and glycerol lead to fat accumulation in the liver (NAFLD) ❖Impaired insulin signaling (IRS-1/PI3K/Akt2)	❖Increased hepatic glucose production ❖Insulin resistance in the liver ❖Disrupted glucose metabolism
5. Ectopic Fat Deposition in Muscle	❖Fat accumulation in skeletal muscle	❖Muscle insulin resistance ❖Elevated circulating VLDL levels ❖Disrupted lipid metabolism in the liver and muscle
6. Chronic Hyperglycemia	❖Insulin resistance leads to elevated blood glucose levels	❖Persistent hyperglycemia ❖Progressive damage to β-cell function and insulin secretion
7. β-Cell Dysfunction and Insulin Secretion Failure	❖Excess fatty acids and glucose lead to β-cell stress ❖Activation of inflammation, ER stress, and oxidative stress	❖Reduced β-cell function and mass ❖Insulin secretion capacity fails
8. Mutual Exacerbation of Disorders	❖Continued inflammation, oxidative stress, and ER stress in β-cells ❖Ongoing hyperglycemia and lipid imbalance	❖Progressive worsening of insulin resistance and β-cell dysfunction ❖Progression to T2DM

In summary, the pathomechanism of T2DM is multifactorial, involving a complex interplay between insulin resistance, beta-cell dysfunction, chronic inflammation, dyslipidemia, and genetic predisposition. These factors disrupt normal glucose metabolism and contribute to the progression of the disease [24]. A deeper understanding of these underlying mechanisms is essential for developing more effective therapeutic strategies to improve the management and outcomes of T2DM.

### 1.3 Diabetic complications and impact of genetic variability on therapeutic efficacy in type 2 diabetes

The National Diabetes Committee has recognized Type 1 Diabetes Mellitus (T1DM), Type 2 Diabetes Mellitus (T2DM), and Gestational Diabetes Mellitus (GDM) as critical health concerns. When left untreated, diabetes can lead to numerous serious health complications affecting both small and large blood vessels—referred to as microvascular and macrovascular complications, respectively. This results in increased morbidity and mortality [25]. One significant complication associated with diabetes is hyperkalemia, a condition characterized by elevated potassium levels in the blood. Hyperkalemia can contribute to various systemic dysfunctions and is often a result of diabetic complications involving both micro- and macrovascular damage. These vascular impairments disrupt normal organ function and may lead to serious outcomes if not properly managed [26]. Among the microvascular issues, kidney damage (diabetic nephropathy) is one of the most costly complications, often progressing to chronic kidney failure. Nerve damage (diabetic neuropathy) increases the risk of diabetic foot ulcers and may ultimately lead to limb amputations. Additionally, eye damage (diabetic retinopathy) is a major cause of blindness. On the other hand, macrovascular complications such as coronary artery disease, peripheral artery disease, and stroke significantly elevate the risk of cardiovascular events and mortality. These complications stem from vascular system impairment combined with chronic hyperglycemia. The body's inability to properly metabolize sugars, proteins, and electrolytes contributes to excessive glucose accumulation in specific cells, dysregulated lipid metabolism, increased oxidative stress, and abnormal cellular growth. As a result, highly vascularized and sensitive tissues such as the retina, renal glomeruli, myocardium, and peripheral nerves are particularly vulnerable, especially the endothelial cells that line their blood vessels [27].

Pharmacogenetics traditionally focuses on single-gene effects on drug metabolism (e.g., *CYP450* enzyme polymorphisms), whereas pharmacogenomics encompasses broader, genome-wide interactions involving multiple genes and environmental factors. Pharmacogenomics offers a promising avenue for improving T2DM management through personalized medicine. By understanding the genetic basis of drug response, clinicians can move toward more targeted, effective, and safer therapies [28]. Continued research, infrastructure development, and policy support will be essential for translating pharmacogenomic advances into clinical practice. Genetic polymorphisms affect the pharmacokinetics (drug absorption, distribution, metabolism, and excretion) and pharmacodynamics (drug action and efficacy) of key antidiabetic drugs [29]. Allele frequencies and drug response phenotypes vary significantly across populations. This highlights the critical need for ethnically diverse genome-wide association studies (GWAS) and functional validations of identified variants. There is a great need for integrating genetic screening into routine diabetes care, especially through preemptive genotyping—an approach in which relevant pharmacogenomic data are obtained before treatment initiation and used to inform drug and dosage selection. The implementation of preemptive pharmacogenomic panels in electronic health records could support clinical decision-making in real time and enhance the precision of T2DM treatment [30].

Variants in *SLC22A1* (OCT1), *SLC22A2* (OCT2), *SLC47A1* (MATE1), and *SLC47A2* (MATE2) alter metformin absorption, distribution, and excretion. For example, *SLC22A1* rs622342 affects hepatic uptake, influencing glycemic control, especially in South Asian and East Asian populations. Similarly, the rs2289669 polymorphism in *SLC47A1* is associated with variations in renal clearance and therapeutic response [31]. In turn, Genetic variants in *ABCC8* (SUR1) and *KCNJ11* (Kir6.2) affect the function of ATP-sensitive potassium channels, modulating drug efficacy and the risk of hypoglycemia [32]. The *CYP2C9* gene, responsible for sulfonylurea metabolism, also shows polymorphisms (*CYP2C9* ∗2 and ∗3) that result in altered drug metabolism, leading to increased drug exposure and potential side effects. Variants such as *CYP2C8* ∗3 and ∗2 affect drug pharmacokinetics and the risk of adverse events [33]. The *PPARG* gene (Pro12Ala variant) is linked with enhanced response to TZDs. Other modifiers include *PPARGC1A* and *ADIPOQ*, which influence insulin sensitivity and metabolic outcomes. Pharmacogenomic research has identified several polymorphisms in *GIPR* and *GLP1R* that impact the efficacy of incretin-based therapies. *GLP1R* rs6923761 and rs10305420 are associated with variations in glycemic response and weight loss outcomes. *TCF7L2*, a well-established diabetes susceptibility gene, also affects incretin hormone function and response to DPP-4 inhibitors. Genetic polymorphisms in *UGT1A9* and *UGT2B4* affect drug metabolism and plasma exposure, particularly for canagliflozin. Variants in *WFS1* (rs10010131) and *PNPLA3* (rs738409) influence weight loss and liver fat reduction with dapagliflozin. Polymorphisms in *SLC5A2* impact blood pressure and glucose-lowering effects of empagliflozin [30]. Despite growing evidence, pharmacogenomics in diabetes care is still emerging. Challenges include the need for large-scale, multiethnic studies to validate findings, clinician education, and regulatory frameworks. As genotyping becomes more accessible and cost-effective, its integration into routine diabetes care could significantly enhance the precision and effectiveness of treatment.

### 1.4 A comprehensive Overview of treatment strategies for type 2 diabetes mellitus

The standard treatment for T2DM primarily aims to regulate blood sugar levels through lifestyle modifications, oral medications, and insulin therapy. Commonly used drugs include metformin, sulfonylureas, and newer agents like GLP-1 receptor agonists and SGLT2 inhibitors. While these treatments are effective, they often have limitations, such as side effects, diminishing efficacy over time, and difficulties maintaining optimal glycemic control [34].

In recent years, advancements in medical research have paved the way for innovative therapies that more effectively address the underlying pathophysiology of T2DM. These include emerging approaches like personalized medicine [35], gene therapies [36], stem cell treatments [37], and the use of advanced technologies such as continuous glucose monitoring [38] and artificial pancreas systems [39]. Additionally, novel pharmacological agents and biological treatments are being investigated to improve patient outcomes, particularly for those resistant to conventional therapies [40].

This article delves into the conventional therapies currently employed in treating T2DM, their mechanisms of action, and the associated challenges. It also explores future perspectives in innovative treatment strategies, highlighting cutting-edge research and potential breakthroughs that could revolutionize the management of this pervasive condition.

## 2 Standard diabetes treatment

The first step of conventional therapy is diet and exercise; antihyperglycemic agents are included (Table 2.). They are distinguished into various classes, either as monotherapy or, more frequently, in combination with one another:

• **Biguanides** – Metformin;
• **Insulin Secretagogues** - Sulfonylureas, Metglinides;
• **Insulin Sensitizers** - Thiazolidinediones (TZDs);
• **Incretin-Based Therapies** - DPP-4 Inhibitors and GLP-1 Agonists;
• **Renal Glucose Transport Modifiers** - SGLT2 Inhibitors;
• **Carbohydrate Absorption Modifiers -** Alpha-Glucosidase Inhibitors;
• **Other Therapeutic Agents** - Bile Acid Sequestrants;
• **Insulin** [41].

**Table 2:** Juxtaposition of glucose-lowering agents.

Class of Drug	Drug Examples	Mechanism of Action	Common Adverse Effects
Biguanides	Metformin [46,[75], [76], [77]]	Reduce hepatic glucose production and increase insulin sensitivity in peripheral tissues.	Gastrointestinal issues (nausea, diarrhea), Lactic acidosis (rare)
Sulfonylureas	Glimepiride [78], Glipizide [79], Glibenclamide (Glyburide) [80,81]	Stimulate insulin secretion from pancreatic beta-cells	Hypoglycemia, Weight gain, Gastrointestinal disturbances
Thiazolidinediones (TZDs)	Pioglitazone [82], Rosiglitazone [83,84]	Increase insulin sensitivity in peripheral tissues by activating PPAR-gamma receptors.	Weight gain, Edema, Bone fractures, Increased risk of heart failure
Dipeptidyl Peptidase-4 (DPP-4) Inhibitors	Sitagliptin [85], Saxagliptin [86], Linagliptin [87], Alogliptin [88]	Inhibit DPP-4 enzyme, Prolong the action of incretin hormones (GLP-1, GIP), which increase insulin secretion and decrease glucagon release	Nasopharyngitis, Headache, Gastrointestinal issues
Glucagon-Like Peptide-1 (GLP-1) Receptor Agonists	Exenatide [89], Liraglutide [90], Dulaglutide [91], Semaglutide (Ozempic®) [92]	Mimic GLP-1, increasing insulin secretion in response to meals, Inhibit glucagon release, Slow down gastric emptying	Nausea, Vomiting, Diarrhea, Risk of pancreatitis (rare)
Sodium-Glucose Cotransporter 2 (SGLT2) Inhibitors	Canagliflozin [93], Dapagliflozin [94], Empagliflozin [95], Ertugliflozin [96]	Inhibit SGLT2 in the kidneys, Reduce glucose reabsorption, and Increase glucose excretion in urine	Urinary tract infections, Dehydration, Hypotension, Genital fungal infections
Alpha-Glucosidase Inhibitors	Acarbose [97], Miglitol [98], Voglibose [99]	Inhibit enzymes in the small intestine that break down carbohydrates and slow glucose absorption.	Flatulence, Diarrhea, Abdominal discomfort
Meglitinides	Repaglinide [100], Nateglinide [101], Mitiglinide [102]	Stimulate rapid, short-term insulin secretion from the pancreas, with a quick onset and short duration of action	Hypoglycemia, Weight gain, Gastrointestinal disturbances
Bile Acid Sequestrants	Colestipol (Colestid) [103]; Cholestyramine (Locholest, Prevalite, and Questran) [104]; Colesevelam (Welchol) [105]	Binds bile acids in the intestines, which may help improve insulin sensitivity, Lower blood glucose	Constipation, Bloating, Gas, Nausea
Insulin	**Rapid-acting insulins** - bolus insulin - Insulin aspart (NovoRapid) Insulin glulisine (Apidra) Insulin lispro (Humalog); **Short-acting insulins** - bolus insulin - Insulin regular (Entuzity, Humulin-R, Novolin ge Toronto); **Intermediate-acting insulins** - basal insulin - Insulin NPH (Humulin-N and Novolin ge NPH); **Long-acting insulins** - basal insulin - Insulin detemir (Levemir) Insulin glargine (Lantus); **Ultra long-acting insulins** - basal insulin **-** Degludec (Tresiba) Insuline glargine (Toujeo) [[106], [107], [108]]	Replaces or supplements insulin that is not produced by the pancreas, Promoting glucose uptake into cells	Hypoglycemia, Weight gain, Injection site reactions, Cardiovascular disease

### 2.1 Metformin – first-line treatment

Lifestyle changes and metformin are the first-line treatments recommended in nearly all guidelines, with a multifaceted mechanism of action that primarily affects glucose production, insulin sensitivity, and intestinal glucose absorption. Metformin activates AMP-activated protein kinase (AMPK) in liver cells, inhibiting the mitochondrial glycerol-3-phosphate dehydrogenase (GPDH) enzyme complex, which is essential for gluconeogenesis. This action reduces glucose production in the liver and helps lower blood glucose levels, particularly during fasting. Additionally, AMPK activation facilitates the translocation of glucose transporter proteins (like GLUT4) to the cell membrane, enhancing glucose entry into the cells. [42,43]. Metformin is especially appropriate for patients with insulin resistance and may reduce cancer-related mortality among individuals with diabetes [44]. In addition to its effects on glucose metabolism, metformin also has beneficial impacts on lipid metabolism. It can lower triglyceride levels and reduce LDL cholesterol, which is particularly advantageous given the heightened cardiovascular risk associated with T2DM. The exact mechanisms that underlie metformin's effects on lipid profiles remain not entirely understood. However, they are believed to be linked to its activation of AMPK and its capacity to enhance insulin sensitivity, indirectly affecting lipid metabolism [45]. Metformin should be initiated at a low dose of 500 mg orally twice daily and increased by 500 mg daily every 1–2 weeks until the patient reaches the maximum tolerated dose. Extended-release tablets are available for those who cannot tolerate the gastrointestinal side effects of the standard immediate-release formulation [46]. If metformin is contraindicated or not well tolerated, sulfonylurea treatment is added; however, it carries a risk of hypoglycemia and cardiovascular issues, especially in elderly and multimorbid patients [47].

### 2.2 Insulin therapy – the cornerstone of diabetes management

Insulin, the primary molecule in diabetes, remains the best treatment even after eighty-five years of use. Individuals with type 1 diabetes mellitus do not produce endogenous insulin, so basal, exogenous insulin administration is crucial for regulating glycogen breakdown, gluconeogenesis, lipolysis, and ketogenesis. Exogenous insulin therapy for patients with T2DM may be necessary in situations such as acute illness or surgery, during pregnancy, to manage glucose toxicity, when oral antidiabetic medications are ineffective or contraindicated, or when a more flexible treatment regimen is needed. In this context, insulin may be administered alone or in combination with oral medications when glycated hemoglobin A1c (HbA1c) is ≥ 7.5 % (≥58 mmol/mol). It becomes vital for those with HbA1c ≥ 10 % (≥86 mmol/mol), especially when diet, physical activity, and other antihyperglycemic treatments have been maximally utilized [48]. The insulin regimen includes multiple daily injections in the abdomen, legs, back of arms, or buttocks by insulin infusion devices (pumps, pens, and syringes) [49]. Differences in categorizing insulins include:

• **Onset** (speed of action),
• **Peak** (time to achieve maximum impact),
• **Duration** (length of effectiveness before diminishing),
• **Concentration** (amount of insulin),
• **Route of delivery** (subcutaneous or intravenous administration),
• **Absorption and elimination.**

There are three main groups of insulins: Fast-acting (Insulin Aspart, Insulin Lispro, Insulin Glulisine) have an onset of action of 5–15 min, a peak effect between 1 and 2 h, and a duration of action lasting 4–6 h; Intermediate-acting (NPH Human Insulin, Pre-Mixed Insulin) have an onset of action of 1–2 h, a peak effect of 4–6 h, and a duration of action exceeding 12 h; and Long-acting insulins (Insulin Glargine, Insulin Detemir) have an onset of action in 1.5–2 h, with a plateau in insulin effect over the next few hours, followed by a relatively flat duration of action lasting 12–24 h [50].

### 2.3 Other conventional treatments

The previously mentioned sulfonylureas are classified as insulin secretagogues. They enhance insulin secretion by binding to receptors on pancreatic β-cells, demonstrating their most significant effect on fasting hyperglycemia. This group includes first-generation agents such as acetohexamide, chlorpropamide, tolazamide, and tolbutamide, as well as second-generation agents like gliclazide, glyburide, and glimepiride [51].

Onward, metglinides (glinides, including mitiglinide, nateglinide, and repaglinide) are non-sulfonylurea insulin secretagogues characterized by a rapid onset and shorter duration of action. They bind to the SUR1 receptors on pancreatic β-cells; however, they have a lower affinity than sulfonylureas and stimulate insulin release [52]. Numerous adverse effects, including nausea, diarrhea, vomiting, constipation, gastrointestinal upset, and abdominal pain, limit their use. Due to their short duration of action, related hypoglycemia is much less persistent with sulfonylureas [53].

Thiazolidinediones (TZDs), also known as “glitazones,” are insulin sensitizers that target intracellular metabolic pathways to enhance insulin activity and improve insulin sensitivity in essential tissues. TZDs reduce both postprandial and fasting hyperglycemia by lowering insulin resistance and increasing the efficiency of endogenous insulin. Furthermore, TZDs lead to higher adiponectin levels, reduced hepatic gluconeogenesis, and increased insulin-dependent glucose uptake in fat and muscle. TZDs may also regulate gene expression by binding to peroxisome proliferator-activated receptor-gamma (PPAR-gamma), which might induce apoptosis in cancer cells [54]. From 2000 to 2008, pioglitazone and rosiglitazone ranked among the most commonly prescribed antidiabetic medications. However, TZDs presented several side effects: weight gain, increased fluid retention, heightened risk of heart attack, and greater likelihood of peripheral fractures [55].

Inhibitors of dipeptidyl peptidase 4 (DPP-4 inhibitors or gliptins) are a class of oral hypoglycemic agents that block the enzyme dipeptidyl peptidase 4 (DPP4). This enzyme is a transmembrane glycoprotein with a molecular mass of 220–240 kDa, initially known as the T cell surface marker cluster of differentiation 26 (CD26) [56]. DPP-4 inhibitors that have been approved by the Food and Drug Administration (FDA) include linagliptin, sitagliptin, saxagliptin, and alogliptin [57]. Vildagliptin is approved by the European Medicines Agency (EMA) [58] but not by the FDA. DPP-4 is a widespread enzyme that affects incretin hormones, mainly GIP (gastric inhibitory peptide) and GLP-1 (glucagon-like peptide-1), which help maintain glucose homeostasis by increasing insulin secretion and reducing glucagon secretion. In addition to its antihyperglycemic effects, this class of drugs displays antihypertensive, anti-inflammatory, and anti-apoptotic effects and immunomodulatory activity in the heart, kidneys, and blood vessels, regardless of the incretin pathway [59].

GLP-1 is a key gastrointestinal hormone that regulates blood glucose levels by increasing insulin secretion from pancreatic β-cells, inhibiting glucagon secretion, slowing gastric emptying, and suppressing appetite by stimulating the satiety center, while also reducing inflammation and apoptosis. GLP-1 significantly decreases insulin resistance and VLDL (very low-density lipoprotein) production through the gut-liver intrinsic signaling pathway. Additionally, GLP-1 exhibits cardio- and neuroprotective effects, influencing learning, memory, and reward-related behaviors [60]. Liraglutide and semaglutide, drugs from the GLP-1 analog group, have demonstrated significant potential in clinical trials, not only in terms of weight loss but also due to their cardioprotective effects [61]. DPP-4 inhibitors are a class of medications that block the enzyme DPP-4, thus extending the action of endogenous GLP-1. By inhibiting DPP-4, these drugs prevent the breakdown of GLP-1, leading to increased levels of GLP-1 and prolonged activity [62]. GLP-1 and GLP-1 receptor agonists effectively treat T2DM by promoting glucose-dependent insulin secretion, inhibiting glucagon secretion, and displaying hypoglycemic properties effects [63]. GLP-1 agonists (also known as GLP-1 receptor agonists, incretin mimetics, or GLP-1 analogs) can be broadly categorized into two main types: human GLP-1 backbone agents (such as Dulaglutide, Liraglutide, and Semaglutide, and the discontinued Albiglutide) and exendin-4 backbone agents (including Exenatide with two formulations, the discontinued Lixisenatide, and Tirzepatide, a GIP analog that activates both the GLP-1 and GIP receptors) [64]. GLP-1 and glucose-dependent insulinotropic polypeptide (GIP), both of which are incretin hormones inactivated by dipeptidyl peptidase-4 (DPP-4), stimulate insulin secretion following an oral glucose load through the incretin effect [65]. Moreover, they inhibit glucagon production from pancreatic α-cells during high blood sugar levels, leading to delayed gastric emptying. Additionally, GLP-1 receptor agonists can reduce pancreatic β-cell apoptosis while promoting their proliferation [66,67].

The next class of antidiabetic drugs consists of sodium-glucose cotransporter-2 (SGLT2) inhibitors, which are taken orally to help reduce hyperglycemia in patients with type 2 diabetes mellitus (T2DM). SGLT2 inhibitors include empagliflozin, ertugliflozin, dapagliflozin, and canagliflozin [68]. SGLT2 inhibitors differ from other antihyperglycemic oral agents due to their insulin-independent mode of action. They lower blood glucose levels through glycosuria and natriuresis by inhibiting glucose reabsorption in the proximal tubule of the kidney [69]. This mechanism stands out from all other glucose-lowering drugs because it does not affect incretin pathways or endogenous insulin [70].

Furthermore, alpha-glucosidase inhibitors (acarbose, miglitol, and voglibose) operate in the intestinal lumen, where these agents competitively inhibit enzymes in the brush border of enterocytes that hydrolyze starches and disaccharides into glucose [71]. Inhibition of α-glucosidase significantly delays carbohydrate absorption after a meal, reducing post-prandial glucose levels. The secretion of gastric inhibitory polypeptide (GIP) is reduced while secretion of glucagon-like peptide-1 (GLP-1) is increased [72].

Bile acid sequestrants (such as colestyramine, colesevelam, colestilan, and colestipol) have improved glycemic control, although their exact mechanisms remain unclear and are likely multifaceted. Possible mechanisms include reducing or slowing carbohydrate absorption, which minimizes postprandial glucose spikes, and decreasing fat absorption, potentially promoting weight loss and enhancing glycemic regulation. Moreover, these agents may affect overall energy metabolism through interactions with the farnesoid X receptor (FXR)—a nuclear receptor involved in bile acid and lipid metabolism—or through FXR-independent pathways that influence the expression of genes regulating glucose and lipid metabolism, such as the suppression of cholesterol 7α-hydroxylase and sterol 27-hydroxylase gene transcription [73,74].

## 3 Innovative/experimental diabetes treatment

In recent years, a range of new antidiabetic medications has been developed, with others undergoing extensive investigation in clinical trials. These emerging therapies help lower blood sugar and target the metabolic components associated with diabetes. Some notable examples include:

• Activin Type II Receptor Modulators (e.g., bimagrumab);
• Amylin or Dual Amylin-Calcitonin Receptor Agonists (e.g., pramlintide-amylin agonist);
• Adenosine Monophosphate-Activated Protein Kinase (AMPK) Activators (e.g., A-769,662, thienopyridone);
• Fibroblast Growth Factor 21 Analogs (e.g., pegbelfermin);
• Fructose-1,6-Bisphosphatase Inhibitors (e.g., VK0612, MB07803);
• New GLP-1 Receptor Agonists (e.g., albiglutide, dulaglutide, exenatide, liraglutide, lyxisenatide, semaglutide, efpeglenatide, glutazumab, ITCA-650);
• Sodium-Glucose Cotransporter (SGLT) Inhibitors (e.g., SGLT1 inhibitors like licogliflozin, sotagliflozin, LX2761; SGLT2 inhibitors like canagliflozin, dapagliflozin, empagliflozin, ertugliflozin, ipragliflozin, luseogliflozin, remogliflozin, tofogliflozin);
• Imeglimin, a novel class of drugs known as glimins.

These therapies are designed to enhance diabetes management by controlling blood glucose and addressing the broader metabolic issues that accompany the disease [96,97]. Table 3 summarizes examples of innovative drugs.

**Table 3:** Summarize of novel/experimental drugs currently being investigated for the treatment of T2DM.

Class of Drug	Drug Examples	Mechanism of Action	Potential Benefits	Side Effects
Dual Incretin Receptor Agonists (GLP-1/GIP)	Tirzepatide (Mounjaro) [198,199]	It combines the GLP-1 receptor agonist and GIP (gastric inhibitory polypeptide) receptor agonist actions to enhance insulin secretion, inhibit glucagon release, and promote weight loss.	Significant improvements in glycemic control and weight reduction.	Gastrointestinal issues (nausea, vomiting), injection site reactions
GIP Receptor Triagonists	Retatrutide [113]	Stimulates GIP receptors to enhance insulin secretion, reduce glucagon secretion, and improve glucose metabolism.	Potential for better glucose control and weight loss.	Gastrointestinal discomfort, headache, injection site reactions
SGLT1/2 Inhibitors (Dual Inhibition)	Sotagliflozin [117,200],	Dual inhibition of SGLT1 (in the intestines) and SGLT2 (in the kidneys) reduces glucose absorption and enhances renal glucose excretion.	Potential for significant blood glucose lowering and improved weight loss.	Diarrhea, dehydration, hypotension, increased risk of UTIs
Glucagon Receptor Antagonists	Bay 27–9955 [123], LY2409021 [124].	Blocks glucagon receptors, reducing hepatic glucose production and lowering blood sugar levels.	Effective in lowering blood glucose, especially in insulin-resistant patients.	Liver enzyme elevations, gastrointestinal disturbances
GPR119 Agonists	DA-1241 [131], ZB-16 (ZB40-0016) [132], JNJ-38431055 [134]	Activates the GPR119 receptor, increasing insulin secretion and improving glucose tolerance.	Potential for better glucose control without weight gain.	Diarrhea, nausea, headache
Fibroblast Growth Factor 21 (FGF21) Analog	Pegylated FGF21 (PEG-cFGF21) [139], Fc-FGF21(RGE) [201], PF05231023 [138], LY2405319 [137]	Mimics the effects of FGF21, regulating glucose and lipid metabolism and improving insulin sensitivity.	Potential to improve insulin sensitivity and reduce visceral fat.	Fatigue, nausea, headache, possible liver enzyme elevation
AMP-activated Protein Kinase (AMPK) Activators	**Indirect:** (metformin) [76], thiazolidinediones **(**troglitazone, pioglitazone, rosiglitazone) [150], polyphenols (resveratrol, quercetin, genistein, epigallocatechin gallate, berberine, curcumin) [151], triterpene glycosides (ginsenoside, Rb1) [152], α-liponic acid (ALA) [153], cryptotanshinone [154], and DNA-damaging agents (cisplatin or metals, including arsenite, vanadate and cobalt through reactive oxygen species ROS activation) [155]. **Direct:** 5-aminoimidazole-4-carboxamide riboside (AICAR) [156], thienopyridone (A-769662) and benzimidazole (Compound 911) derivatives [146], salicylate (pro-drug of Asprin) [77], AMP mimetics (5-(5-hydroxyl-isoxazole-3-yl)-furan-2-phosphonic acid [157], termed Compound-2 (C-2), and its pro-drug C-13) [158], PT-1 [159], MT 63–78 (Debio0930) [160]	It activates AMPK, promoting glucose uptake and fatty acid oxidation and reducing hepatic glucose production.	Improved insulin sensitivity and glucose metabolism.	Gastrointestinal side effects, fatigue
Bromodomain and Extra-Terminal (BET) Inhibitors	RVX-208 [164,165], ABBV-744 [167]	Inhibits BET proteins that regulate gene expression involved in inflammation and metabolism, improving insulin sensitivity and reducing inflammation.	Potential to reduce insulin resistance and inflammation in T2DM.	Infections, fatigue, gastrointestinal symptoms
Thymosin Beta-4 Analog	TB-500 [172], The actin-sequestering protein thymosin beta-4 (Tβ4) [173]	Regulates glucose homeostasis and enhances pancreatic beta-cell function.	Possible improvement in beta-cell regeneration and glucose control.	Injection site reactions, mild gastrointestinal effects
Monogenic Therapy (Gene Editing)	CRISPR-Cas9 [179], ZFNs [186,187], TALENs [192,193]	Gene-editing technology that aims to correct mutations associated with beta-cell function or insulin resistance.	Potential for long-term, permanent correction of diabetes-related gene defects.	Ethical concerns, potential off-target effects, immune responses
Proprotein Convertase Subtilisin/Kexin Type 9 (PCSK9) Inhibitors	monoclonal antibodies (Evolocumab, Alirocumab, Bococizumab, and Tafolecimab), small interfering RNA (siRNA, Inclisiran), adnectin BMS-962476 [197,202]	Inhibits PCSK9, leading to increased insulin sensitivity and improved lipid metabolism.	Potential for improved glucose control, particularly in diabetic dyslipidemia.	Injection site reactions, muscle pain, fatigue

### 3.1 Dual and triple receptor agonists: advancing diabetes treatment with tirzepatide and retatrutide

Tirzepatide (LY3298176) is the leading dual GIP/GLP-1 receptor agonist, designed as a single-molecule treatment for T2DM. It comprises a 39-amino acid synthetic peptide linked to a C20 fatty diacid moiety [109]. Acylation with fatty acids is a well-established technique in the diabetes field, previously used to extend insulin and GLP-1's duration of action. This modification allows the peptide to bind to albumin, increasing its biological half-life. It is administered via a once-weekly subcutaneous injection and has recently received approval from the European Medicines Agency [110]. GLP-1 promotes insulin secretion after meals while simultaneously inhibiting glucagon release, both in a glucose-dependent manner. Additionally, it enhances feelings of fullness by acting on the central nervous system (specifically the hypothalamus) and slowing gastric emptying, which helps reduce hunger. GIP is an incretin hormone that boosts insulin secretion in response to food intake [111]. Unlike GLP-1 and GLP-1 receptor agonists, which are well-known for inhibiting glucagon secretion, the effects of GIP on glucagon release are more complex and not yet fully understood. The underlying mechanisms remain debated; some suggest that GIP directly influences glucagon secretion, while others propose an indirect effect by enhancing GLP-1's appetite-suppressing actions. In addition to its role in glucagon regulation, GIP offers benefits to peripheral tissues, including adipose tissue and skeletal muscle. It promotes lipid storage in white adipose tissue while reducing fat accumulation in muscle tissue, ultimately improving insulin sensitivity [112]. Therefore, GIP and GLP-1 demonstrate complementary and potentially synergistic effects, rendering their combination a promising strategy for enhancing glucose regulation and facilitating weight loss. A dual GIP/GLP-1 receptor agonist has been suggested as a “super GLP-1 receptor agonist” due to GIP's ability to enhance GLP-1's actions, possibly leading to greater therapeutic benefits in managing T2DM and obesity [112].

Onwards, retatrutide (LY3437943) is a once-weekly single peptide that functions as a triple receptor agonist targeting the G-protein–coupled receptors: GIP, GLP-1, and glucagon (GCG) receptors. It exhibits greater potency at human GIP receptors than native hormones and is less effective at human glucagon and GLP-1 receptors. In preclinical studies, retatrutide reduced food intake and increased energy expenditure, likely due to its action on the glucagon receptor. In a multiple-ascending dose trial involving individuals with T2DM, retatrutide showed significant reductions in both glucose levels and body weight [113]. The mechanism of action of retatrutide may also involve oxyntomodulin, amylin, and peptide YY receptors, which appear to influence the regulation of body fat mass and energy homeostasis [114].

### 3.2 Targeting SGLT1/2 for glycemic control and cardiorenal benefits: advances in dual inhibition therapy in diabetes and its related complications

Inhibiting SGLTs offers a relatively new therapeutic strategy for enhancing glycemic control and has also been proven to provide cardiorenal benefits. Dual SGLT1/2 inhibitors (SGLT1/2i), such as sotagliflozin, target both SGLT1 and SGLT2 proteins, delivering a broader mechanism of action for managing diabetes and its related complications [115]. Rieg et al. (2014) demonstrated that both genetic and pharmacological inhibition of SGLT2 results in increased SGLT1-mediated glucose reabsorption in the kidney under euglycemic conditions. This heightened activity of SGLT1 helps to offset the decreased glucose reabsorption due to SGLT2 inhibition, thus maintaining glucose homeostasis [116]. Animal studies on sotagliflozin have demonstrated myocardial benefits through dual SGLT1/2 inhibition in normoglycemic mice subjected to cardiac pressure overload. These cardiac improvements occurred without changes in whole-body or cardiac-specific metabolism regarding fatty acid or ketone body utilization. Instead, the advantages were mainly attributed to significant diuresis and glucosuria. However, the absence of myocardial benefits in high-fat diet (HFD) mice indicates that proximal tubular injury may have compromised the drug's cardioprotective effects [117].

T2DM significantly worsens heart failure (HF) outcomes, contributing to increased mortality, hospitalization rates, and reduced response to therapies such as cardiac resynchronization therapy (CRT). As reported by Sardu et al. [118], elderly diabetic patients receiving CRT demonstrated a diminished likelihood of treatment response compared to non-diabetic counterparts. This impaired response is largely attributed to the metabolic and inflammatory derangements associated with DM, including chronic hyperglycemia, oxidative stress, and endothelial dysfunction. Mechanistically, DM promotes excessive production of reactive oxygen species (ROS) and pro-inflammatory cytokines, contributing to myocardial fibrosis, impaired contractility, and adverse cardiac remodeling—factors that undermine CRT efficacy [119].

Sodium-glucose cotransporter 2 inhibitors (SGLT2i), initially developed as antidiabetic agents, have demonstrated cardioprotective effects beyond glycemic control. SGLT2i reduce oxidative stress, inhibit NF-κB activation, and improve mitochondrial efficiency, thereby limiting myocardial damage. Moreover, SGLT2i enhance antioxidant defenses, reduce fibrosis, and improve endothelial function. These actions collectively support improved cardiac performance and HF outcomes, independent of diabetic status [120]. Sardu et al. proved that metformin therapy in pre-diabetes patients undergoing coronary artery bypass grafting for acute myocardial infarction significantly reduces inflammatory markers, SGLT2, and leptin levels, while increasing SIRT6 expression, leading to improved cardiovascular outcomes. Pre-diabetes patients not using metformin had higher rates of major adverse cardiac events and elevated pericoronary fat inflammatory markers compared to normoglycemic patients. [121]. SGLT2 inhibitors significantly reduce inflammation and improve long-term clinical outcomes in patients with T2DM undergoing coronary artery bypass grafting with minimally invasive extracorporeal circulation. These findings support the use of SGLT2 inhibitors as an adjunctive therapy to enhance recovery and reduce complications in diabetic patients after coronary artery bypass grafting (CABG).

Importantly, the integration of SGLT2i into the treatment of diabetic HF patients may improve CRT responsiveness by mitigating the detrimental effects of hyperglycemia and inflammation. Thus, SGLT2i represent a promising adjunct therapy in HF management, particularly for patients with comorbid diabetes [122].

### 3.3 Glucagon receptor antagonists and GPR119 agonists: emerging therapeutic targets in T2DM

Glucagon receptor antagonists (GRAs) represent a novel class of therapeutic agents that target the glucagon receptor (GCGR), a G-protein-coupled receptor (GPCR) playing a central role in glucose homeostasis. This class includes agents such as Bay 27–9955 [123] and LY2409021 [124]. Glucagon, secreted by pancreatic alpha cells, binds to glucagon receptors (GCGRs) primarily in the liver, stimulating gluconeogenesis and glycogenolysis, which increases hepatic glucose production, particularly during fasting or hypoglycemic conditions. In type 2 diabetes mellitus (T2DM), elevated glucagon levels contribute to persistent hyperglycemia by promoting excessive hepatic glucose output, even in the presence of high insulin levels. Glucagon receptor antagonists (GRAs) work by selectively binding to GCGRs, blocking their activation by endogenous glucagon, and preventing the downstream signaling that drives glucose production. By inhibiting glucagon receptor signaling, GRAs reduce gluconeogenesis and hepatic glucose release, thus lowering blood glucose levels. Notably, because glucagon's effects are glucose-dependent, blocking its receptor typically does not cause hypoglycemia, making GRAs a potentially safer alternative to insulin or sulfonylureas, which carry a risk of insulin-induced hypoglycemia [125,126]. Additionally, some GRAs have shown extra benefits in reducing body weight and regulating lipid metabolism, which is crucial for managing obesity-related T2DM. Some GRAs have also demonstrated the capacity to decrease hepatic fat accumulation and enhance non-alcoholic fatty liver disease (NAFLD), a common comorbidity in people with T2DM [127].

GPR119 (G protein-coupled receptor 119, glucose-dependent insulinotropic orphan receptor) has recently emerged as a promising target for T2DM treatment. GPR119 is located in pancreatic β-cells, intestinal L-cells, and other tissues, including the liver. When activated, GPR119 stimulates increased insulin secretion and improved blood sugar control [128,129]. GPR119 primarily signals by activating cyclic AMP (cAMP), which is mediated through the stimulation of the Gαs protein subunit [130]. While several agonists of GPR119 have been developed and tested in early clinical trials, they proved unsuccessful in phase II due to a loss of effectiveness over time and tachyphylaxis. In contrast, DA-1241 has shown enduring antidiabetic effects in diabetic animal models, as highlighted in previous studies. It has been demonstrated to reduce blood glucose levels without tachyphylaxis and to enhance glucose and lipid metabolism, positioning DA-1241 as a potentially effective treatment for T2DM [131]. Tyurenkov et al. demonstrated that the novel GPR119 agonist ZB-16 (1 mg/kg) effectively reduces fasting blood glucose levels in animals with experimental T2DM induced by streptozotocin and nicotinamide. Treatment with ZB-16 also enhanced glucose utilization, as indicated by improved results on the glucose tolerance test, which are consistent with Glucagon receptor antagonists (GRAs). Furthermore, ZB-16 administration resulted in an increased percentage of insulin-positive pancreatic endocrine cells. These effects were associated with elevated secretion of GLP-1 and insulin, suggesting that ZB-16 operates through an incretin-like mechanism [132]. JNJ-38431055 is an orally bioavailable, selective GPR119 agonist developed from a series of GPR119 activators identified by Semple et al. [133]. In a study involving healthy male adults, JNJ-38431055 was well tolerated and, compared to a placebo, significantly increased post-meal levels of active and total GLP-1, gastric insulinotropic peptide (GIP), and peptide YY (PYY). These findings confirmed that JNJ-38431055 elicited the expected pharmacological effects of a GPR119 agonist in healthy individuals. The current research represents the first investigation of JNJ-38431055 in hyperglycemic diabetic patients, utilizing standard oral glucose tolerance tests (OGTT) and mixed meal tolerance tests (MMTT) to assess its pharmacodynamic effects. The study evaluates the impact of single oral doses on glucose levels, insulin secretion, and incretin release in a placebo-controlled, positive-controlled crossover trial. Additionally, a parallel-design trial examines the effects of 14-day oral administration of JNJ-38431055 on 24-h weighted mean plasma glucose and incretin release compared to placebo. The current research represents the first investigation of JNJ-38431055 in hyperglycemic diabetic patients, utilizing standard oral glucose tolerance tests (OGTT) and mixed meal tolerance tests (MMTT) to assess its pharmacodynamic effects. The study evaluates the impact of single oral doses on glucose levels, insulin secretion, and incretin release in a placebo-controlled, positive-controlled crossover trial. Additionally, a parallel-design trial examines the effects of 14-day oral administration of JNJ-38431055 on 24-h weighted mean plasma glucose and incretin release compared to placebo [134]. In summary, preclinical studies primarily focus on the effects of GRAs and GPR119 agonists in animal models, while clinical studies explore the effects of these agents in human trials to assess their safety, efficacy, and impact on metabolic parameters like blood glucose, insulin secretion, and incretin release.

### 3.4 Fibroblast growth factor 21 (FGF21): A promising target for diabetes, obesity, and metabolic disease treatment

Fibroblast growth factors (FGFs) are crucial in various metabolic processes. Within the endocrine subgroup, FGF19, FGF21, and FGF23 have shown potential therapeutic benefits for diabetes mellitus, and obesity. FGF21 is the most promising because it can lower blood glucose levels, increase insulin sensitivity, and support weight loss in animal models. FGF21 has emerged as a key therapeutic target for metabolic diseases, including non-alcoholic steatohepatitis (NASH), metabolic syndrome, and DM. It improves insulin sensitivity, facilitates glucose uptake in adipocytes, and supports weight loss, particularly in obesity and diabetes models. Unlike insulin, FGF21 does not cause hypoglycemia or weight gain, making it a potentially safer alternative or complementary therapy to existing treatments [135].

In preclinical studies, FGF21 has shown significant metabolic benefits, including reductions in plasma glucose and triglyceride levels without causing hypoglycemia. It enhances glucose uptake in adipose tissue by upregulating GLUT1, a crucial glucose transporter, and inhibits hepatic gluconeogenesis, thereby lowering blood glucose levels. Additionally, FGF21 promotes the differentiation of stem cells into adipocytes and boosts lipid oxidation, which helps reduce liver steatosis and improve insulin sensitivity. It also increases energy expenditure by activating brown adipose tissue (BAT), facilitating fat oxidation and thermogenesis, contributing to weight loss. These combined effects emphasize FGF21's potential as a therapeutic agent for metabolic disorders [136].

Several FGF21 analogs have been developed to improve the stability and pharmacokinetics of the protein, as native FGF21 is biophysically unstable and unsuitable for clinical use. These analogs include LY2405319 [137], PF05231023 [138], and PEG-cFGF21 [139]. LY2405319, a stabilized FGF21 variant, has been tested in mice, monkeys, and humans in clinical studies, demonstrating improvements in lipid profiles with reductions in triglycerides and LDL cholesterol. In animal models, it has also been shown to induce weight loss and reduce blood glucose levels without causing hypoglycemia. A trend toward normoglycemia was observed in humans, although more research is needed to fully understand the effects on insulin sensitivity and β-cell function [137].

Another FGF21 analog, PF05231023, has shown similar metabolic benefits, including reductions in triglycerides, HDL levels, and weight loss in monkeys and humans in clinical studies. However, human studies did not demonstrate a significant effect on blood glucose levels. Some side effects were reported, including diarrhea, bone resorption, and increased heart rate and blood pressure. PF05231023 exhibited promising potential for improving lipid metabolism and body composition despite these limitations. Further optimization is necessary to enhance its therapeutic profile while minimizing adverse effects [138].

PEG-cFGF21, a PEGylated version of canine FGF21, has been tested in diabetic dogs, where it showed effectiveness in lowering glucose levels and improving lipid profiles. Compared to insulin, PEG-cFGF21 maintained glucose control for extended periods (up to 3 days) and enhanced liver GLUT1 expression, which benefits glucose regulation. This approach has demonstrated promise for treating diabetic pets, and further studies are needed to evaluate its potential in cats and other animals [139].

Despite elevated FGF21 levels in obese and diabetic individuals, evidence suggests these patients may resist FGF21 signaling. This could explain why the increased FGF21 levels in these individuals do not fully reverse metabolic disturbances. FGF21 resistance may be linked to the reduced expression of its receptors (β-klotho and FGFR1) in adipose tissue and the liver, particularly in conditions such as obesity and after prolonged ketogenic diets [140].

In conclusion, FGF21 and its analogs show significant promise for managing metabolic diseases such as diabetes mellitus (DM) and obesity. These therapies can enhance insulin sensitivity, lower hyperglycemia, regulate lipid metabolism, and promote weight loss. However, challenges persist, including the potential for resistance in obese individuals and the necessity to address side effects like gastrointestinal issues and increased blood pressure. Future research should concentrate on optimizing the pharmacological properties of FGF21 analogs, comprehending their mechanisms across different species, and investigating their CRT effects. The development of FGF21-based therapies could provide a novel approach to treating diabetes, potentially in combination with insulin or other therapeutic strategies, and even extend to veterinary medicine for managing animal diabetes [135].

FGF21 analogs have shown promise in treating metabolic conditions such as obesity, T2DM, and NASH, largely due to their effects on improving insulin sensitivity, lipid profiles, and energy expenditure. However, the long-term impacts of sustained FGF21 receptor activation are not fully understood. While these analogs are engineered for enhanced stability and potency, they may interact with other fibroblast growth factor receptors, leading to off-target metabolic or endocrine effects [141]. Additionally, preclinical studies have raised concerns about possible adverse effects on bone density and the central nervous system, which have not yet been comprehensively evaluated in human trials [142]. Most available data come from early-phase trials with limited sample sizes and follow-up durations. Therefore, the broader safety profile of FGF21 analogs, especially with chronic administration, remains to be established through larger, long-term studies. Careful post-marketing surveillance will be essential to detect delayed or rare adverse effects and to assess sustained efficacy in real-world settings [141].

### 3.5 AMP-activated protein kinase: mechanisms, activators, and therapeutic potential in metabolic disorders and cancer

Subsequently, AMP-activated protein kinase A (AMPK) is a serine/threonine protein kinase complex that consists of a catalytic α-subunit (α1 and α2), a scaffolding β-subunit (β1 and β2), and a regulatory γ-subunit (γ1, γ2, and γ3) [143]. AMPK regulates the balance between anabolic and catabolic processes to maintain cellular homeostasis under metabolic stress. It is significant in glucose and lipid balance, body weight regulation, food intake, insulin signaling, and mitochondrial biogenesis [144]. AMPK is considered a key target for treating cancers and metabolic disorders like T2DM and obesity. It acts as a cellular energy sensor, getting activated under various conditions that diminish cellular energy, such as nutrient deprivation (mainly glucose), hypoxia, and exposure to toxins that disrupt the mitochondrial respiratory chain complex [145]. Direct AMPK activators consist of small molecules that mimic AMP, leading to a conformational change in AMPK and enabling further activation through the phosphorylation of Thr-172 on the AMPKα subunit. Recent structural studies have shown that the cystathionine-β-synthase domain repeats in the AMPKγ subunit are crucial to this process. Sites 1 and 3 regulate the binding of AMP, ADP, or ATP. Binding of AMP to Site 1 initiates allosteric activation, while binding AMP or ADP to Site 3 influences Thr-172 phosphorylation. Although ADP is more plentiful than AMP, AMP remains the primary activator that boosts LKB1-dependent phosphorylation. This binding induces a conformational shift, activating the AMPK complex [146,147]. AMPK plays a critical role in maintaining cellular homeostasis by balancing anabolic and catabolic processes in response to metabolic stress. It is involved in the regulation of glucose and lipid balance, body weight, insulin signaling, and mitochondrial function, making it a significant target for treating metabolic diseases such as T2DM and obesity. The connection between AMPK's role in tumorigenesis and LKB1, an upstream kinase and tumor suppressor, is notable. Activating AMPK with drugs like metformin has been shown to delay tumor formation. AMPK mediates tumor-suppressive pathways through mTORC1, TIF-1A, and cell cycle arrest involving p53, p21, and p27kip1. However, once tumors are established, AMPK may assist cancer cells in surviving under harsh conditions, potentially complicating its application in cancer therapy. Additionally, AMPK regulates autophagy, which helps maintain cellular balance by recycling components. It phosphorylates key proteins such as ULK1 and PI3KC3/VPS34, influencing processes like glycogenolysis and lipolysis. Understanding AMPK's role in autophagy could offer further insights into its effects on metabolism. Given its extensive functions, significant efforts are being directed toward developing AMPK modulators for treating various diseases. This review summarizes the potential therapeutic applications of AMPK activators [148]. AMPK activation also involves phosphorylation at Thr-172 by the upstream kinases LKB1 and CaMKKβ. AMP binding enhances LKB1-dependent phosphorylation and inhibits dephosphorylation by phosphatases. Myristoylation of the AMPKβ subunit's N-terminus is also necessary for this effect. In contrast, CaMKKβ activates AMPK in response to increased Ca2+ levels without altering AMP levels, making AMP increase AMPK's susceptibility to phosphorylation. Thus, the regulation of AMPK entails a complex interplay between AMP binding, phosphorylation, and subunit modifications [149]. AMPK can be activated directly and indirectly by AMP or calcium accumulation. Indirect AMPK activators include biguanides (metformin) [76], thiazolidinediones **(**troglitazone, pioglitazone, rosiglitazone) [150], polyphenols (resveratrol, quercetin, genistein, epigallocatechin gallate, berberine, curcumin) [151], triterpene glycosides (ginsenoside, Rb1) [152], α-liponic acid (ALA) [153], cryptotanshinone [154], and DNA-damaging agents (cisplatin or metals, including arsenite, vanadate and cobalt through reactive oxygen species ROS activation) [155]. Direct activators activate AMPK without significantly changing cellular ATP, ADP, or AMP levels. They comprise 5-aminoimidazole-4-carboxamide riboside (AICAR) [156], thienopyridone (A-769662) and benzimidazole (Compound 911) derivatives [146], salicylate (pro-drug of Asprin) [77], AMP mimetics (5-(5-hydroxyl-isoxazol-3-yl)-furan-2-phosphonic acid [157], termed Compound-2 (C-2), and its pro-drug C-13) [158], PT-1 [159], and MT 63–78 (Debio0930) [160]. Hither, preclinical studies are primarily discussed, with some clinical applications mentioned (e.g., metformin).

### 3.6 Targeting BET bromodomains: implications for diabetes, obesity, and cardiovascular disease therapy

Next, the BET (Bromodomain and Extra-Terminal) family of epigenetic reader proteins—comprising BRD2, BRD3, BRD4, and BRDT (collectively referred to as BETs)—modulates gene transcription by recognizing and binding to acetylated histones. Epigenetic modulation involves the addition (writing) or removal (erasing) of histone modifications on chromatin, which facilitates changes in heterochromatin, enabling its transition to the open, activated euchromatin state necessary for transcription [161]. Hu et al. demonstrated that a deficiency in BRD4 expression in myeloid lineage-specific cells protects mice from obesity, inflammation, and insulin resistance caused by a high-fat diet, especially within their adipose tissues [162]. Wang et al. discovered that disrupting BRD2 led to the development of severe obesity; the knockout mice exhibited normal glycemia and glucose tolerance, as well as lower levels of inflammation in their adipose tissues compared to the control mice. Whole-body disruption of Brd2, a unique MHC gene, causes severe lifelong obesity in mice, accompanied by pancreatic islet expansion, hyperinsulinemia, hepatosteatosis, and elevated pro-inflammatory cytokines. Surprisingly, these mice also show enhanced glucose tolerance, elevated adiponectin levels, increased brown adipose tissue mass, heightened heat production, and upregulated mitochondrial uncoupling proteins in brown adipose tissue. Additionally, there is reduced macrophage infiltration in white adipose tissue and lowered blood glucose, resulting in an improved metabolic profile and protection from eventual T2DM. Brd2 is highly expressed in pancreatic β-cells, where it typically suppresses β-cell mitosis and insulin transcription. In 3T3-L1 pre-adipocytes, Brd2 co-represses PPAR-γ (peroxisome proliferator-activated receptor-γ) and inhibits adipogenesis. Brd2 knockdown in these cells protects against TNF-α (tumor necrosis factor-α)-induced insulin resistance, thereby decoupling inflammation from insulin resistance. Thus, hypomorphic Brd2 shifts the energy balance toward storage without causing glucose intolerance, providing a potential model for obese, metabolically healthy humans [163].

Recent randomized clinical trials have investigated the effects of BET inhibitors on diabetes and related conditions [161]. In one trial, RVX-208 was evaluated in unmedicated males with prediabetes using an oral glucose tolerance test to assess postprandial glucose levels, insulin sensitivity, glucose kinetics, and lipolysis. The results indicated that RVX-208 produced a similar initial plasma glucose peak as the placebo but showed a more sustained elevation 30 min later. Furthermore, RVX-208 treatment reduced and delayed glucose secretion while suppressing endogenous glucose production. This resulted in a lower glucose disappearance rate, although it did not impact glucose oxidation or total glucose disposal. These findings suggest that RVX-208 may delay and reduce glucose absorption and endogenous production, potentially protecting the development of T2DM [164].

In another trial, RVX-208 was assessed throughout 3–6 months to evaluate its effects on lipid parameters, coronary atherosclerosis, and the occurrence of major adverse cardiovascular events (including death, myocardial infarction, and coronary revascularization). The findings indicated that patients treated with RVX-208 experienced fewer cardiovascular events, especially those with diabetes, than the placebo group, suggesting a potential benefit for heart health [165].

The phase III BETonMACE trial further examined RVX-208's effects in 2425 patients with recent acute coronary syndrome (ACS) and diabetes. Treatment with RVX-208 resulted in fewer hospitalizations for heart failure, along with a decrease in the total number of heart failure hospitalizations and combined cardiovascular death or hospitalization. These findings indicate that RVX-208 may be especially beneficial in reducing heart failure-related hospitalizations among patients with T2DM and ACS [166]. Together, these trials suggest that inhibiting BET could provide substantial clinical benefits for patients with diabetes, such as enhanced glucose regulation and decreased cardiovascular risks. Together, these trials suggest that inhibiting BET could provide substantial clinical benefits for patients with diabetes, such as enhanced glucose regulation and decreased cardiovascular risks [161]. Another BET bromodomain inhibitor with selectivity for the second bromodomain is *N*-Ethyl-4-[2-(4-fluoro-2,6-dimethyl-phenoxy)-5-(1-hydroxy-1-methyl-ethyl)phenyl]-6-methyl-7-oxo-1*H*-pyrrolo[2,3-*c*]pyridine-2-carboxamide (ABBV-744) [167]. Further BET inhibitors used in oncological and cardiovascular indications include GSK973 [168], OTX-015, Ten-010, I-BET762, CPI-610, ABBV-075, BMS-986158, INCB054329, INCB057643, and PLX51107 [167]. Preclinical studies have focused on BRD4 and BRD2 gene disruptions in animal models, examining their effects on obesity, insulin resistance, and glucose metabolism. These studies have provided insights into how these BET family proteins influence metabolic processes and offer potential therapeutic targets for conditions like obesity and T2DM. In contrast, clinical studies have investigated RVX-208 in human patients, evaluating its effects on diabetes, cardiovascular events, and related conditions. These trials have shown promising results in improving glucose regulation and reducing cardiovascular risks, particularly in individuals with diabetes.

### 3.7 Thymosin β4 and its analogs: potential therapeutic strategies for diabetic complications and tissue regeneration

Thymosin β4 (Tβ4), a polypeptide with a molecular weight of 5 kDa comprised of 43 amino acids, is the most abundant member of the β-thymosin family in mammalian tissues and is recognized as the primary peptide involved in sequestering G-actin. Tβ4 plays a crucial role in promoting wound healing and tissue repair, accelerating both dermal and corneal wound healing and enhancing heart function after a myocardial infarction. Additionally, Tβ4 supports neuron survival and the growth of neurites in cultured spinal cord neurons. Its angiogenic properties further facilitate endothelial cell (EC) migration, tubule formation, and angiogenesis. Tβ4 also aids in differentiating epicardial progenitor cells into ECs, contributing to the formation of vascular progenitors for coronary vasculogenesis and angiogenesis. Currently, the role of Tβ4 in diabetic ECs is still poorly understood. To date, it has been shown that Tβ4 enhances the migration of CD34+/KDR + circulating endothelial progenitor cells (EPCs) in diabetic fatty rats [169]. The study by Zhu et al. aimed to evaluate the effectiveness of Tb4 in managing hyperglycemia and enhancing insulin sensitivity in a mouse model of T2DM. KK mice were divided into several groups: the KK control group, which received saline treatment, and the KK Tb4 group, which received daily Tb4 injections (100 ng/10 g body weight) for 12 weeks. Non-diabetic C57BL mice served as the standard control. Various measures, including the oral glucose tolerance test (OGTT), plasma insulin, HbA1c, serum adiponectin, Tβ(4), cholesterol, and triglycerides, were collected before and after Tb4 treatment. Additionally, the levels of phosphorylated AKT and total AKT proteins in skeletal muscle were analyzed.

After Tb4 treatment, the repeat OGTT indicated a significant reduction in glucose levels in the KK Tb4 group compared to the KK control group. The KK Tb4 group also displayed lower HbA1c and triglyceride levels and higher adiponectin than the KK control group. C57BL mice maintained normal glucose regulation. Additionally, the levels of phosphorylated AKT in skeletal muscle were significantly higher in the KK Tb4 group than in the KK control group after glucose stimulation. In contrast, no changes in phosphorylated AKT levels were noted in the C57BL mice following Tb4 treatment. These results suggest that Tb4 improved glucose intolerance and alleviated insulin resistance in the KK mouse model of T2DM. Hence, Tb4 may be a promising insulin sensitizer for treating T2DM [170]. Typically, Tβ4 analogs are synthetic or modified forms of Tβ4 created to enhance their therapeutic properties. These analogs are often developed to improve stability and bioavailability or target specific functional effects, such as promoting wound healing, tissue repair, or enhancing angiogenesis [171]. TB-500 is among the most widely used and researched synthetic analogs of Tβ4. It consists of the N-terminal acetylated 17–23 amino acids of Tβ4. Known for its wound-healing properties, it enhances cell migration and tissue repair [172]. The actin-sequestering protein thymosin beta-4 is another synthetic peptide related to Tβ4. It mimics the actin-sequestering activity of Tβ4, helping regulate the dynamics of actin filaments in cells. It is critical for cellular and physiological processes, including cell migration, proliferation, motility, growth, metastasis, and wound healing. In diabetes, it promotes the regeneration of pancreatic cells [173]. Some studies have involved creating Tβ4 derivatives with minor modifications to the amino acid sequence to improve their stability or effectiveness. These modifications include slight sequence alterations or conjugation to other molecules to target specific tissues or increase bioavailability [174,175]. The information provided here focuses on preclinical data.

### 3.8 Novel approaches for diabetes treatment: cell-based therapies and genetic engineering innovations

A promising cell resource for diabetes treatment is human pluripotent stem cell-derived islets (hPSC-islets), which contain nearly pure populations of endocrine cells [176]. Unlike conventional iPSCs, which are created by overexpressing transcription factors in somatic cells, CiPSCs (Chemical-induced Pluripotent Stem Cells) are produced using small-molecule chemicals as reprogramming factors. These chemicals are easy to manufacture, non-genomic, scalable, and tunable, providing a more standardized approach. This method offers an alternative means to generate human pluripotent stem cells (hPSCs) that may be more suitable for therapeutic applications [177]. After differentiation into β cells, corrected SC-β cells should exhibit robust dynamic insulin secretion in vitro in response to glucose, similar to WFS1 in iPSCs derived from a patient with Wolfram syndrome (WS) [178].

CRISPR/Cas9 systems (clustered regularly interspaced short palindromic repeats-associated Cas protein system) evolved as part of prokaryotes' adaptive immune response to defend against bacteriophages, invading plasmids, and viruses. In the bacterial genome, A–T-rich leader sequences are adjacent to 27–42 base pair palindromic repeats, which are separated by proto-spacers. These spacers are segments of DNA derived from previously encountered bacteriophages, serving as a molecular record of past infections. The CRISPR system utilizes these protospacers as templates to recognize and defend against subsequent viral invaders. [179]. CRISPR/Cas9 technology is increasingly used to manipulate human pluripotent stem cells (hPSCs) to model monogenic diseases (MDs) and study pancreatic development. These technologies enable the creation of gene-edited hPSCs to explore mutations associated with MDs, particularly those impacting pancreatic development and beta-cell function [180]. Several protocols for differentiating hPSCs into pancreatic cell types have been developed, including those that generate definitive endoderm and posterior foregut-like populations. This advancement facilitates the study of diseases such as MODY3, where mutations in genes like HNF1A affect pancreatic cell differentiation. CRISPR-Cas9 editing can introduce specific genetic changes in hPSCs to model these conditions. The general steps involved in this process include designing sgRNAs, synthesizing them, transfecting hPSCs, assessing Indel frequency, and propagating knockout lines. Notable studies have employed CRISPR/Cas9 to investigate pancreatic transcription factors like PDX1, RFX6, PTF1A, GLIS3, MNX1, NGN3, HES1, and ARX [181]. For instance, Wang et al. investigated how mutations in PDX1 and related factors influence pancreatic cell commitment, demonstrating how low PDX1 levels affect beta-cell function and development [182]. Similarly, Cardenas-Diaz et al. utilized CRISPR to ablate HNF1A in ESCs, discovering that it interrupted beta-cell development [183]. Shi et al. examined GATA6 mutations, demonstrating that haploinsufficiency in GATA6 impacts beta-cell differentiation [184], Chia et al. found that GATA6's cooperation with EOMES/SMAD2/3 is essential for proper endoderm development in humans [185]. These approaches provide valuable insights into the genetic basis of diabetes and have the potential to improve disease modeling and therapeutic strategies. Zinc-finger nucleases (ZFNs) are artificial proteins that combine a DNA-binding zinc-finger domain with a non-specific FokI endonuclease to target specific DNA sequences. The zinc-finger domains recognize distinct three-base pair DNA sequences, while the FokI domain dimerizes to produce double-strand breaks (DSBs) in the DNA [186]. When DSBs occur, the DNA damage response is triggered to repair the breaks. The process includes: 1) Introducing ZFNs containing FokI and DNA-binding domains into cells, 2) These domains enter the nucleus, 3) The DNA-binding domains target the sequence to be cleaved, 4) FokI creates the break at the target site, and 5) The desired DNA segment is inserted and integrated into the genome [187]. For gene-editing platforms such as CRISPR-Cas9, many of the demonstrated effects—including precise gene correction and phenotypic improvements—have been validated primarily in murine or other non-human models. Translational applicability to human physiology remains uncertain, particularly in light of species-specific differences in immune responses, gene regulation, and long-term safety profiles. Similarly, GPR119 agonists have shown favorable outcomes in rodent models, including enhanced incretin secretion and improved glycemic control; however, human studies have yielded more variable results, potentially due to differences in receptor distribution and downstream signaling pathways [188]. While CRISPR-Cas9 has revolutionized genome editing by allowing precise modifications of genetic material, concerns remain about its clinical application, especially regarding long-term safety. Despite ongoing improvements in guide RNA specificity and delivery systems, the risk of off-target effects—unintended modifications in non-target genes—persists. Such alterations may disrupt essential gene functions or activate oncogenes, potentially leading to severe or delayed consequences, including tumorigenesis. Furthermore, the delivery of CRISPR components, often via viral vectors or nanoparticles, carries the risk of immune reactions and tissue-specific toxicities [189]. Most clinical studies conducted thus far involve small, homogeneous cohorts, making it difficult to assess how these therapies will perform across genetically and environmentally diverse populations. Importantly, the irreversible nature of genome editing amplifies the importance of long-term surveillance, as adverse outcomes may emerge years after treatment [190]. These uncertainties highlight the need for rigorous post-treatment monitoring and broader studies to evaluate both efficacy and safety across different demographic and clinical contexts [191].

Transcription activator-like effector nucleases (TALENs) have a modular structure, consisting of an N-terminal DNA-binding domain (TALE) fused to a C-terminal FokI endonuclease. Compared to ZFNs, TALENs provide greater specificity and fewer off-target effects because TALE repeats can target single base pairs, while zinc fingers recognize DNA triplets. The TALE DNA-binding domain comprises 34-residue repeat units, with positions 12 and 13 (repeat variable residues, RVDs) interacting with the target DNA. Common RVDs include NI for adenine, HD for cytosine, NG for thymine, and NN/HN for guanine or adenine. TALENs are designed to recognize sequences of 12–20 base pairs, with longer sequences enhancing editing precision. These arrays are typically assembled using Golden Gate cloning. Recent advances in TALENs focus on improving their performance, such as discovering new RVDs to boost activity. The *Fok*I cleavage domain facilitates dimerization and cleaves within a 12–19 base pair spacer between TALE binding sites. The resultant DNA double-strand breaks are repaired by non-homologous end joining (NHEJ) or homology-directed repair (HDR) when a donor template is available [192,193]. The studies described so far in this subsection have focused on preclinical studies.

Finally, proprotein convertase subtilisin/kexin type 9 (PCSK9) is a protein present in the bloodstream that binds to the LDL receptor (LDLR), causing its breakdown in lysosomes. Treatment with anti-PCSK9 monoclonal antibodies (MAbs) can reduce plasma LDL cholesterol (LDL-C) levels by 50–60 % [194]. In clinical studies, the plasma levels of PCSK9 are elevated in patients with T1DM or T2DM, linking PCSK9 to glucose homeostasis. Furthermore, PCSK9 inhibitors reduce the risk of developing cardiovascular disease. The increased plasma PCSK9 levels seen in patients with T2DM may result from the condition's selective hepatic insulin resistance and hypertriglyceridemia [195]. In this state, insulin is ineffective at decreasing hepatic gluconeogenesis through the FoxO1 pathway but still activates hepatic lipogenesis via the SREBP1c pathway [196]. The introduction and clinical approval of novel therapies that inhibit PCSK9 (PCSK9-iTs), including three monoclonal antibodies (Evolocumab, Alirocumab, and Tafolecimab) and a small interfering RNA (siRNA, Inclisiran), represent a significant advancement in the treatment of metabolic diseases [197].

## 4 Conclusions

The management of T2DM has significantly evolved over the years from a one-size-fits-all model to a multifaceted approach integrating metabolic, genetic, and molecular perspectives with a wide array of therapeutic options available to control blood glucose levels and enhance patient outcomes. Conventional therapies, including metformin, sulfonylureas, thiazolidinediones, and insulin, have proven effective in many instances, offering various mechanisms of action to address the underlying causes of T2DM, such as insulin resistance and beta-cell dysfunction. These medications aid in controlling blood glucose, reducing complications, and improving the quality of life for patients. However, the limitations of these drugs, including side effects like hypoglycemia, weight gain, and diminished efficacy over time, highlight the need for more personalized and targeted treatment approaches.

Notably, incretin-based therapies, including GLP-1 receptor agonists and DPP-4 inhibitors, have bridged the gap between glycemic control and weight management. The advent of dual (GLP-1/GIP) and triple (GLP-1/GIP/glucagon) receptor agonists, such as tirzepatide and retatrutide, represents a major pharmacological milestone. These agents not only optimize glucose regulation but also exhibit pronounced effects on appetite suppression and energy homeostasis, paving the way for integrated diabetes-obesity (diabesity) therapies.

The emergence of SGLT2 and dual SGLT1/2 inhibitors underscores a paradigm shift toward insulin-independent glucose-lowering mechanisms. Beyond glycemic control, these agents deliver cardiorenal benefits, particularly in patients with heart failure and chronic kidney disease—common comorbidities in T2DM. This multifaceted utility signifies a crucial step toward holistic patient management.

Experimental therapies such as glucagon receptor antagonists, GPR119 agonists, FGF21 analogs, and AMPK activators further enrich the therapeutic arsenal. These agents target upstream metabolic dysfunctions and systemic inflammation, potentially offering disease-modifying effects. AMPK activators, in particular, highlight the interface between metabolism and oncology, given their dual role in energy regulation and tumor suppression.

Genomic approaches, including CRISPR/Cas9 and TALENs, herald a future where monogenic defects can be corrected at their source. While still in experimental stages, these tools offer unprecedented precision in disease modeling and therapeutic development. The ability to generate patient-specific iPSCs or CiPSCs and differentiate them into β-like cells opens avenues for cell replacement therapies, especially for patients with severe β-cell dysfunction.

Furthermore, BET inhibitors and Thymosin β4 analogs illustrate the rising interest in epigenetic and regenerative medicine, respectively. These agents could help mitigate chronic inflammation and restore tissue integrity, which are crucial for halting or reversing diabetes complications.

Despite the promise of these innovations, several challenges remain that must be addressed before these therapies can be widely adopted into clinical practice. One significant concern is the lack of long-term safety and efficacy data for many of the novel agents, particularly those with pleiotropic effects, such as FGF21 analogs. While early results are promising, the potential for unforeseen adverse events over extended treatment periods warrants cautious evaluation through large-scale, long-term clinical trials. Additionally, gene-editing and stem-cell-based therapies face substantial regulatory hurdles due to their complexity, ethical considerations, and potential off-target effects. These factors can delay development and approval, limiting their immediate clinical applicability. Moreover, the high cost of advanced treatments and technologies, such as continuous glucose monitoring systems, personalized gene therapy, and cell-based regenerative approaches, may pose barriers to widespread implementation, especially in resource-limited settings. Accessibility and affordability are critical issues that must be addressed to ensure equitable healthcare delivery. Another important challenge is the variability in individual responses to these therapies. Differences in genetics, lifestyle, disease stage, and comorbidities mean that a one-size-fits-all approach is unlikely to be effective. This variability underscores the urgent need for reliable biomarkers and precision medicine tools to guide therapeutic selection and optimize outcomes for each patient.

Addressing these challenges through ongoing research, policy reform, and interdisciplinary collaboration will be essential to fully realize the potential of emerging therapies in transforming the future of T2DM care. Furthermore, metformin is widely recognized as one of the most cost-effective drugs for managing T2DM, given its low price, broad availability, and proven effectiveness. Insulin remains essential for T1DM and advanced T2DM, but its higher cost and injection requirements can impact both accessibility and adherence. On the other hand, innovative therapies like GRAs, GPR119 Agonists, and FGF21 analogs are still in the early stages of development or clinical trials, meaning their cost-effectiveness and adherence remains uncertain. Biologic drugs, in general, tend to be significantly more expensive than established treatments like metformin, due to the complexity of their production and the costs associated with their development and distribution.

While these emerging therapies are still under investigation, and their long-term safety and efficacy are yet to be fully established, the future of diabetes treatment appears promising. Discussions have primarily centered on the social, bioethical, and legal implications of using genome editing technologies in human cells. While there is broad consensus among scientists that CRISPR-Cas9 should be permitted for creating human disease models and for advancing our understanding of disease development and molecular mechanisms, its use for eugenics or human enhancement remains widely opposed. Social, legal, and bioethical issues should be thoroughly addressed once genome editing technologies have achieved an acceptable level of safety for clinical applications aimed at preventing genetic diseases with a detailed guide for future processing and use of this technology.

Continued research into novel therapies, coupled with a deeper understanding of the pathophysiology of T2DM, will undoubtedly lead to more effective, personalized, and safer treatment options, ultimately enhancing the quality of life for individuals with T2DM. Taken together, the trajectory of T2DM treatment is moving toward precision medicine, where therapies are tailored not only to glycemic profiles but also to metabolic phenotype, genetic background, and comorbid conditions. Multidisciplinary collaboration between endocrinologists, pharmacologists, molecular biologists, and data scientists will be key to translating these scientific advances into clinical success. While conventional therapies remain vital, the integration of innovative pharmacological, genomic, and regenerative strategies is poised to redefine T2DM management—shifting the paradigm from symptom control to true disease modification.

## CRediT authorship contribution statement

**Barbara Gieroba:** Writing – review & editing, Writing – original draft, Validation, Project administration, Formal analysis, Data curation, Conceptualization. **Adrianna Kryska:** Visualization, Investigation. **Anna Sroka-Bartnicka:** Supervision, Funding acquisition.

## Declaration of generative AI and AI-assisted technologies in the writing process

While preparing this work, the authors used ChatGPT to improve language and readability. After using this tool/service, the authors reviewed and edited the content as needed and took full responsibility for the publication's content.

## Funding

This review has been written as part of a grant from the National Science Centre, project SONATA, “Synergy of chemical imaging methods in diabetic model” (project UMO-2020/39/D/ST4/01604) and within the statutory activity of the Medical University of Lublin (DS 642 project).

## Declaration of competing interest

The authors declare that they have no known competing financial interests or personal relationships that could have appeared to influence the work reported in this paper.

## Data Availability

No data was used for the research described in the article.
